# Novel prognostic signature unveils PSEN1 contributes to depression-induced lung adenocarcinoma progression

**DOI:** 10.3389/fimmu.2026.1681306

**Published:** 2026-01-29

**Authors:** Qiaoqi Zheng, Ji Zhuoga, Congcong Li, Wenjing Chen, Maimaititusun Yalikun, Peng Fu, Zaiquan Dong, Jingcheng Dong

**Affiliations:** 1 Department of Integrative Medicine, Huashan Hospital, Fudan University, Shanghai, China; 2 Institute of Integrative Medicine, Fudan University, Shanghai, China; 3 Mental Rehabilitation Centers, Karamay Municipal People’s Hospital, Xinjiang, Karamay, China; 4 Mental Health Center, West China Hospital, Sichuan University, Chengdu, Sichuan, China

**Keywords:** depression, immunity, lung adenocarcinoma, PSEN1, therapeutic response

## Abstract

**Purpose:**

Depression is acknowledged to correlate with the occurrence and progression of multiple cancers. However, no study has yet systematically complied depression-related genes to construct a prognostic signature for lung adenocarcinoma (LUAD).

**Methods:**

Our study encompasses 1,276 LUAD patients from three cohorts. Consensus clustering was employed to classify patients into different depression subtypes. Then, a variety of machine-learning algorithms were utilized to construct a robust depression-related signature (DRS). Thereafter, a nomogram combining DRS with common clinical characteristics was established for prognosis. The IOBR package was used to quantify the immune landscape, whereas the oncoPredict and Connectivity Map algorithms were employed to evaluate therapeutic response. The Seurat package was applied to process single-cell data, and the Scissor algorithm was used to identify depression-associated cells. Ultimately, depression-like mouse models were constructed to detect alternations in depression-related genes. *In vitro* experiments were performed to explore the role of PSEN1 in the malignant behaviors of LUAD.

**Results:**

Unsupervised clustering stratified patients into two subtypes with distinct features. DRS consisting of 14 hub depression-related genes was established using the LASSO + GBM algorithm and served as an independent prognostic indicator. The nomogram constructed with DRS demonstrated robust predictive efficacy, with a C-index of 0.778. LUAD patients in the high-risk group exhibited weaker “immune hot” features and reduced responsiveness to immunotherapy. Additionally, high-risk patients were less sensitive to conventional chemotherapy and targeted therapies. Single-cell analysis revealed that depression-associated high-risk cells displayed more malignant characteristics. Finally, qRT-PCR validated the alternations of depression-related genes in depression-like mouse models, and *in vitro* experiments confirmed that PSEN1 facilitated cell proliferation in LUAD.

**Conclusions:**

The molecular profile defined by the DRS can serve as an independent overall survival predictor and improve individualized treatment and clinical decision for LUAD patients. Of which, PSEN1 may contribute to depression-induced LUAD progression.

## Introduction

As the most frequently diagnosed malignant cancer worldwide, lung cancer is also the leading cause of cancer-related death (1). In 2022, approximately 2.5 million new cases were confirmed, with about 1.8 million patients dying from the disease (1). As the main histologic subtype of lung cancer, the incidence of lung adenocarcinoma (LUAD) has surpassed that of lung squamous cell carcinoma and continues to rise (2). The prognosis of LUAD correlates with multiple risk factors, particularly depression (3–8). On the one hand, a diagnosis of depression is associated with a higher likelihood of developing cancer (9). On the other hand, a cancer diagnosis often coincides with depression and anxiety disorders, especially death anxiety (10). Depression significantly shortens the survival time of LUAD patients (3, 7, 8). A prospective observational study of 227 patients revealed that emotional distress, including depression, is an independent predictor for worse overall survival and progression-free survival in advanced lung cancer (8). The co-occurrence of depression also reduces the effectiveness of antitumor treatments including chemotherapy (11), radiotherapy (12), and immunotherapy (8). Yue Zeng et al. reported a significant decline in the objective response rate to immune checkpoint inhibitors in patients with concurrent depression (8). Moreover, depression leads to a poorer quality of life in patients with lung cancer (8, 13). Lung cancer patients with concurrent depression exhibit lower global health scores, as well as reduced scores across multiple functional domains, including physical, role, emotional, cognitive, and social functioning (8). Therefore, it is of great importance to elucidate the mechanisms by which depression exerts adverse effects on LUAD.

In this study, we aimed to establish a depression-related signature (DRS) for overall survival prediction. The immune landscape and therapeutic responses of different groups stratified by risk score were further investigated. Finally, experimental validation was performed with depression-like mouse models and *in vitro* experiments.

## Materials and methods

### Cell culture

Lewis lung cancer cells and human LUAD cell lines A549 and H1299 were purchased from the Cell Bank of Chinese Academy of Sciences (Shanghai, China) and maintained in Ham’s F-12K or RPMI-1640 medium (Gibco, Grand Island, NY, United States) supplemented with 10% fetal bovine serum (FBS; 40131ES76, Yeasen Biotechnology), penicillin (100 µg/mL) and streptomycin (100 µg/mL) (Sigma-Aldrich, USA) at 37°C in an atmosphere of 5% CO_2_.

### Animals

Male C57BL/6 J (6–8 weeks old) and BALB/c-nu/nu mice (4–6 weeks old) were purchased from Shanghai Jihui Experimental Animal Feeding Co., Ltd (Shanghai, China) [SCXK (沪) 2022-0009] and raised under specific-pathogen-free conditions at Fudan University (Shanghai, China). Each set was kept in plastic cages (300×200×120 mm) with paper chips for bedding. Rooms were kept at a constant temperature (23 °C ± 3°C) and humidity (50% ± 10%), with simulated day/night cycles (12 h each day, lights on at 8:00 am). Animal experimental protocols were approved by Fudan University’s Institutional Animal Care and Use Committee (Number: 202405FD0001). All authors are informed and agree.

After a 1-week adaption, the BALB/c-nu/nu mice were randomly divided into three groups: NC + Control, PSEN1^OE^ + Control, and PSEN1^OE^ + Alpelisib. In short, 5×10^5^ A549 cells, overexpressed with PSEN1 (PSEN1^OE^) or not (NC), were subcutaneously implanted in the right flank of each mouse in 0.1 mL of PBS. Every 3 days, tumor volumes were measured with a caliper and computed as (length × width × width)/2. After 1 week of injection, Alpelisib (HY-15244, MedChemExpress, China) was prepared with 5% NMP and 95% PEG-300 and then orally administered to mice once a day (50 mg/kg) for 7 consecutive days.

### Chronic restrain stress

After a 1-week adaption, the C57BL/6 J mice were randomly divided into two groups: the control group (Con) and the chronic restrain stress group (CR). In short, 5×10^5^ LLC cells were subcutaneously implanted in the right flank of each mouse in 0.1 mL of PBS. Every 3 days, tumor volumes were measured with a caliper and computed as (length×width×width)/2. Mice in the CR group received 3 h of restraint stress treatment every day for 21 days, from 10:00 to 13:00. During this period, each CR mouse was fixed with a plastic holder without food and water.

### Forced swimming test

The forced swimming test (FST) was used to estimate the depression-related behavior as described before (14). The mice were put inside a plexiglass cylinder that was 200 mm high by 140 mm in diameter and filled with water, which was 21°C or slightly warmer, to a height of 10 cm, preventing the mice from climbing out or touching the bottom. The automatic forced swimming test system (Bioseb BIO-FST-DSM) was used to record for 6 min, of which the immobility time of mice for the last 4 min was analyzed.

### Open field test

The open field test (OFT) was performed in a box (80×80×80 cm). Each mouse was placed in a corner at the start of the test and recorded for 6 min by a camera located above the box. We cleaned the device with 75% alcohol after each trial. A tracking system with an automated analysis system recorded the number of entries into the center zone, the time spent in the center zone, and the total distance traveled (Omnitech SuperFlex). The center area of the open field apparatus was 25% of the total area (a square of approximately 40×40 cm).

### Light–dark box test

For the light–dark box test (LDBT), each mouse was placed in the center of a light compartment (30×30×30 cm, white surfaces) connected with a dark compartment (20×30×30 cm, black surfaces) by a square aperture. Mouse activity was recorded during a 6-min test period. The time that the mouse spent in the light box and the number of transitions from the light to the dark compartment were analyzed by Noldus EthoVision XT.

### RNA isolation and quantitative real-time PCR

Reverse transcription-polymerase chain reaction and real-time PCR (qRT-PCR) were performed according to the manufacturers’ instruction. Briefly, total RNAs were isolated by RNA Extraction Kit (AG21017, Accurate Biology). The cDNAs were obtained by the PrimeScript RT Reagent Kit (RR037A, Takara) and subjected to qRT-PCR with SYBR^®^ Premix Ex Taq (DRR041A, Takara). The relative expression levels of mRNAs compared with GAPDH were calculated by the 2^−ΔΔCt^ method. The primers used in this study are shown in **Supplementary Table 1**.

### Immunofluorescence

Immunofluorescent staining was conducted on tumor tissue sections embedded in paraffin, following established protocols. After standard procedures for deparaffinization, rehydration, and blocking, the sections were subjected to overnight incubation at 4 °C with PSEN1 antibody (1:500, 16163-1-AP, Proteintech, China). Subsequent to PBS washing, the sections were incubated for 1 h at room temperature with fluorescence-conjugated anti-rabbit antibodies (1:100, ZF0516, ZSGB-BIO, China). Following an additional wash, the sections were mounted using a mounting medium containing 4′,6-diamidino-2-phenylindole (DAPI). Ultimately, the stained sections were examined and documented through a confocal microscope. Fluorescence intensity of slices was analyzed by ImageJ software.

### Plasmid transfections

The full length of PSEN1 was amplified and cloned into pcDNA3.1. A549 and H1299 cells were transfected with an empty vector or the PSEN1-overexpression plasmid for 48 h in 12-well plates at 80% confluency according to the manufacturer’s protocol. The transfection efficiency of PSEN1 was validated by qRT-PCR and western blot.

### Western blot

The protein extraction kit (KeyGEN Biotech) was used to extract protein from cells. The specific steps are as follows: add 10 µL phosphatase inhibitor, 1 µL protease inhibitor, and 10 µL PMSF to each mL of cold Lysis Buffer, and place the cells in the mixed lysate and centrifuge. The supernatant is the extracted protein. The protein concentration of each sample was calculated using the BCA method. Proteins were separated by SDS-PAGE electrophoresis and transferred to a polyvinylidene fluoride membrane (Millipore, Boston, Massachusetts, USA). After being sealed with 5% skimmed milk (Sangon Biotech (Shanghai) Co., Ltd.) at room temperature for 1 h, the samples were incubated with Anti-PSEN1 (16163-1-AP, Proteintech, China), Phospho-ERBB1 (Tyr1197) (84906-1-RR, Proteintech, China), ERBB1 (18986-1-AP, Proteintech, China), Phospho-AKT (Ser473) (66444-1-Ig, Proteintech, China), AKT (10176-2-AP, Proteintech, China), and β-Actin (20536-1-AP, Proteintech, China) antibody on a 4°C shaking table overnight and then incubated with horseradish peroxidase bound secondary antibody (1:1,000, Beyotime Biotechnology) at room temperature for 1 h. The protein expression in each sample was analyzed using ImageJ software (NIH, Bethesda, Maryland, USA).

### Cell counting Kit-8 assay

Cell growth was measured by a Cell Counting Kit-8 (Dojindo, Japan). Cells (4 × 10^3^/well) were seeded into a 96-well plate (Corning, New York, USA) and incubated for 1 or 2 days. Cells were twice rinsed gently with PBS, and then we added 10 μL Cell Counting Kit-8 (CCK-8) reagent into each well and incubated the cells at 37°C for 1 h. The absorbance was determined at a wavelength of 450 nm using a microplate reader (Molecular Devices, CA, USA).

### Colony formation assay

In the colony formation assay, 1,000 transfected cells per well were seeded in 12-well microplates and then cultured for 14 days. Colonies were fixed with 4% paraformaldehyde for 10 min, stained with crystal violet (G1062, Solarbio, China) for 10 min, washed repeatedly with water, and counted manually.

### 5-Ethynyl-20-deoxyuridine staining assay

Cells seeded on 96-well plates for 5-ethynyl-20-deoxyuridine (EdU) staining experiment (C10310, RiboBio, China) were incubated with 50 μM EdU for 2 h, followed by incubation of Hoechst 33342 for an additional 30 min to stain the nucleus. The EdU-positive rate was accessed as the ratio of EdU-positive cells to all Hoechst 33342-positive cells in each field.

### Apoptosis assay

According to the instructions of the apoptosis assay kit (C1062S, Beyotime Biotechnology, China), A549 cells were collected and stained with Annexin V and PI and incubated at room temperature in the dark for 20 min, and then the level of cell apoptosis was detected by flow cytometry.

### Enzyme-linked immunosorbent assay

After the animal experiment, serum samples from mice were obtained and the effect of CR on levels of adrenocorticotropic hormone (ACTH, RK09105, ABclonal Technology, China) and corticosterone (COR, RK09054, ABclonal Technology, China) was detected according to the instructions of the enzyme-linked immunosorbent assay (ELISA) kit.

### Data acquisition

RNA-Seq, single-nucleotide variation (SNV), copy number variation (CNV), and clinical information of LUAD samples in TCGA were obtained from the Genomic Data Commons (GDC, https://portal.gdc.cancer.gov). Transcriptome profiling and clinical information of LUAD samples in GSE31210 and GSE72094 were acquired from the GEO database (https://www.ncbi.nlm.nih.gov/geo). The Maftools package was performed to analyze SNV data, whereas GISTIC2.0 was used to process CNV data. Genes related to depression were obtained from the GeneCards database (www.genecards.org/), MSigDB database (www.gsea-msigdb.org/gsea/index.jsp), and previous reported depression-related genes by large cohort consisting of 135,458 cases and 344,901 controls (15). The single-cell RNA-sequencing dataset GSE127465 was acquired from the GEO database. The protein abundance of PSEN1 was accessed from CPTAC (16). Representative images of immunohistochemistry of PSEN1 were acquired from the HPA database (17).

### Consensus clustering

Unsupervised consensus clustering was conducted with the ConsensusClusterPlus package based on 17 depression-related genes (DRGs). Principal component analysis (PCA) was performed with the scatterplot3d package to depict the differences between two subgroups. Survival and survminer packages were used to verify the prognostic value whereas KEGG and GSEA analyses were utilized for pathway enrichment.

### Establishment of prognostic depression-related signature

Leveraging various combinations of machine learning algorithms (GBM, RSF, SuperPC, survival-SVM, Lasso, stepCox, Ridge, Enet, CoxBoost, and plsRcox), we constructed a depression-related signature based on the LASSO + GBM algorithm, which performed moderately in both training set TCGA-LUAD and test sets GSE31210 and GSE72094, illustrated by the C-index. Survival analyses were performed with the survival package, PCA was performed with the scatterplot3d package, and receiver operating characteristic curves were carried out with the timeROC package. The differences of risk score between different clinicopathologic subgroups were calculated with two independent sample t-tests, whereas the stratified survival analyses were performed to validate the prognostic value of DRS in diverse subpopulations including age, gender, clinical stage, T stage, N stage, and M stage. Univariate Cox regression and multivariate Cox regression was utilized to verify the prognostic capacity of DRS for overall survival.

### Construction and assessment of DRS-based nomogram

R package “rms” was applied for the construction of DRS-based nomogram to predict overall survival for 1/3/5 years. Then, we calculated the C-index with the calibration curve and performed ROC to assess the prognostic value of the nomogram.

### Estimation of immune landscape

Estimate, MCPCOUNTER, EPIC, TIMER, and quantiseq algorithms were wielded with the IOBR package, whereas single sample gene set enrichment analysis (ssGSEA) was performed with the GSVA package to access the infiltration of immune cells. Then, immune subtypes were calculated with the ImmuneSubtypeClassifier package. In addition, TIDE scores were calculated with the Tumor Immune Dysfunction and Exclusion database (https://tide.dfci.harvard.edu) whereas immunophenoscore (IPS) was obtained from The Cancer Immunome Atlas (TCIA) database (https://tcia.at/home). Additionally, the correlation between risk score and immune cell infiltration, immune checkpoints, immune pathways, or TIDE score were performed with Pearson’s correlation analyses.

### Calculation of therapeutic response

Drug sensitivity to chemotherapy and specific targeted therapy was calculated using the oncoPredict package. Differential expression analysis was processed with the limma package. Then, Connectivity Map (CMap) analysis was performed with the CLUE database (https://clue.io).

### Scissor algorithm

Harnessing both bulk data and phenotypic information, the Scissor algorithm facilitates the selection of cell subpopulations from single-cell sequencing that are primarily contributed to the different phenotypes. Utilizing transcriptomics data of diverse depression phenotypes in TCGA-LUAD, we used the Scissor algorithm to associate each cell in GSE127465 with its most probably corresponding phenotype. Rigorous QC was applied to the raw single-cell data. Cells were retained only if they expressed more than 200 genes and had a mitochondrial gene ratio below 10%. Cells with a high hemoglobin gene expression (percent.HB > 1%) were also filtered out to remove potential red blood cell contaminants. Following QC, the data were normalized using the LogNormalize method. Cell types were annotated using a standardized workflow based on canonical marker genes. Briefly, after clustering, cell type annotation was performed in a two-step manner, combining coarse classification (**Supplementary Figure 1E**) followed by fine-grained subdivision (**Supplementary Figure 1F**), based on canonical marker gene expression as follows: CD4+ T cells (CD4, CD28, CD3D, IL7R), CD8+ T cells (CD8A, CD8B, CCL5, GZMB), NK cells (FGFBP2, GNLY, KLRC1, KLRD1), B cells (CD19, MS4A1, CD79A), plasma cells (MZB1, SDC1, IGKC, SLAMF7), Mono/Macro cells (CD14, CSF1R, CD68, TYROBP), DC cells (CD1C, CD86, CD1A), mast cells (CPA3, TPSAB1, MS4A2), neutrophil cells (S100A9, S100A8, IL1R2), endothelial cells (VWF, CDH5, CLDN5), and fibroblasts (COL1A2, COL6A2, DCN, COL1A1). In the Scissor algorithm, we set the parameters as alpha = 0.005 and beta = 0.2.

### Statistical analysis

All above analyses were conducted in R v4.5.0 and GraphPad Prism v10.1.2. The differences between the low- and high-risk groups were compared with Student’s t-test or Wilcoxon rank-sum test. All statistical tests if not specified were two-sided, and p-value < 0.05 was considered to be statistically significant.

## Results

### Identification of 17 hub prognostic depression-related genes in LUAD

**Figure 1:**
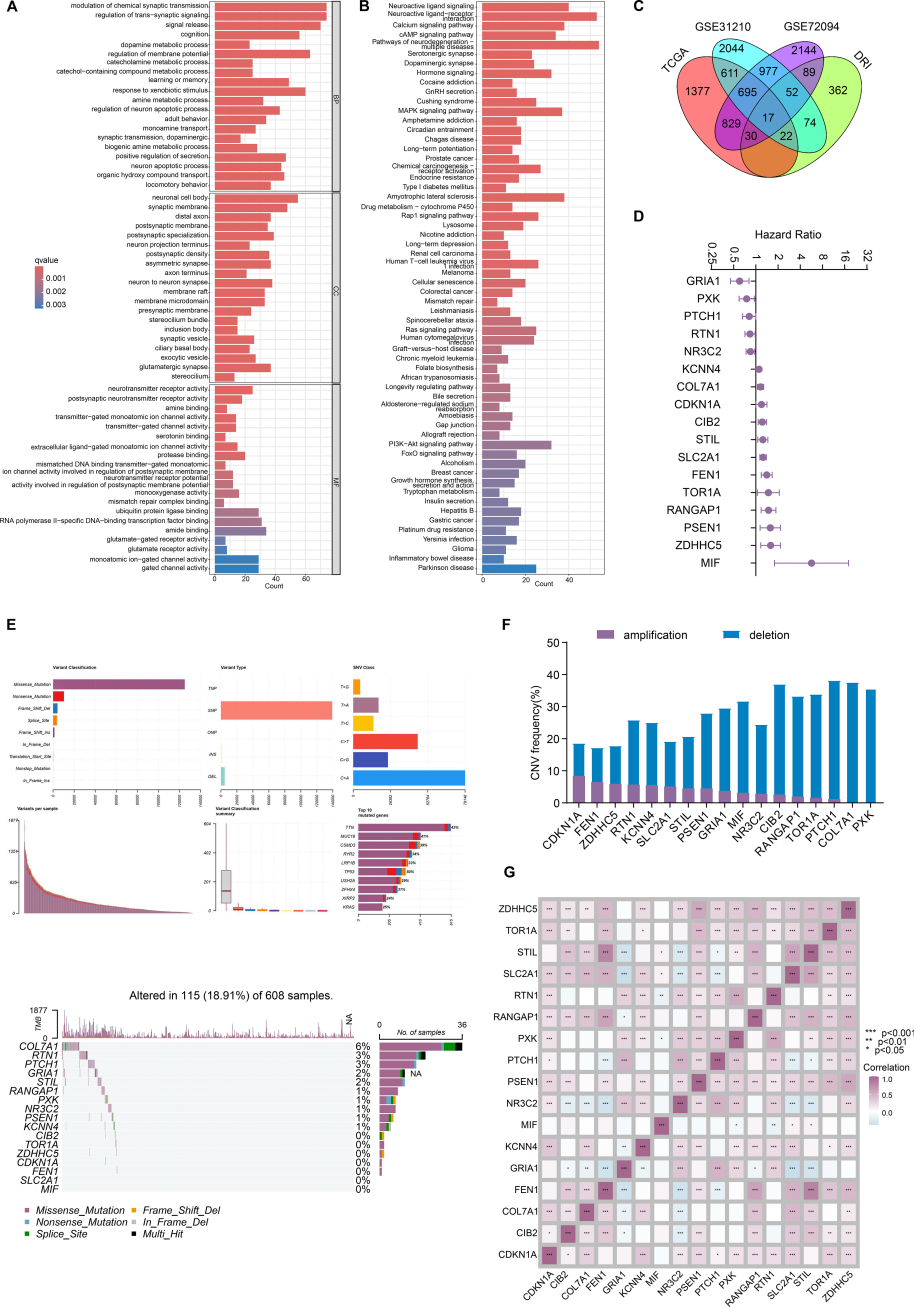
Identification of 17 hub depression-related genes. **(A)** GO analysis revealed the gene ontology of 692 depression-related genes we collected; **(B)** KEGG analysis demonstrated the involved signaling of 692 depression-related genes; **(C)** 17 prognostic depression-related genes (DRGs) were overlapped across three cohorts; **(D)** univariate Cox regression indicated the hazard ratios of 17 hub DRGs; **(E)** landscape of SNV of 17 hub DRGs; **(F)** landscape of CNV of 17 hub DRGs; **(G)** correlation heatmap unveiled the initial relationship among 17 hub DRGs. *P < 0.05, **P < 0.01, and ***P < 0.001.

We obtained 692 depression-related genes from the GeneCards database (www.genecards.org/), MSigDB database (www.gsea-msigdb.org/gsea/index.jsp), and previous reported depression-related genes from a large cohort study consisting of 135,458 cases and 344,901 controls (15). Then, we conducted Gene Ontology (GO) enrichment analysis on these depression-related genes, which revealed their involvement in biological processes (BP) such as “modulation of chemical synaptic transmission,” “regulation of trans−synaptic signaling,” “cognition,” and “dopamine metabolic process”; cellular components (CC) including “neuronal cell body,” “synaptic membrane,” and “distal axon”; molecular functions (MF) such as “neurotransmitter receptor activity” and “postsynaptic neurotransmitter receptor activity,” among others (**Figure 1A**). Consistently, KEGG analysis confirmed that these genes were enriched in pathways including “Neuroactive ligand signaling” (18, 19), “Neuroactive ligand−receptor interaction” (18, 20), “Dopaminergic synapse” (21, 22), and “Hormone signaling” (23, 24), which have been reported to be closely related to the occurrence of depression (**Figure 1B**). To identify prognostically relevant genes, we intersected the three cohorts (TCGA-LUAD, GSE31210, and GSE72094) with the above depression-related genes based on their largest sample size (**Figure 1C**) and identified 17 overlapped depression-related genes (DRGs) using univariate Cox regression analysis (**Figure 1D**). Furthermore, these 17 DRGs exhibited distinct levels of single-nucleotide variations (SNVs), particularly missense mutations, with an overall mutation frequency of 18.91% in the TCGA-LUAD (**Figure 1E**). Similarly, varying degrees of copy number variation (CNVs) were also observed among these genes (**Figure 1F**), which may partially account for their prognostic significance. Thereafter, correlation analysis was performed to explore the potential relationships among these genes, revealing both positive and negative correlations (**Figure 1G**).

### Consensus clustering divides patients into two depression-related subtypes

**Figure 2:**
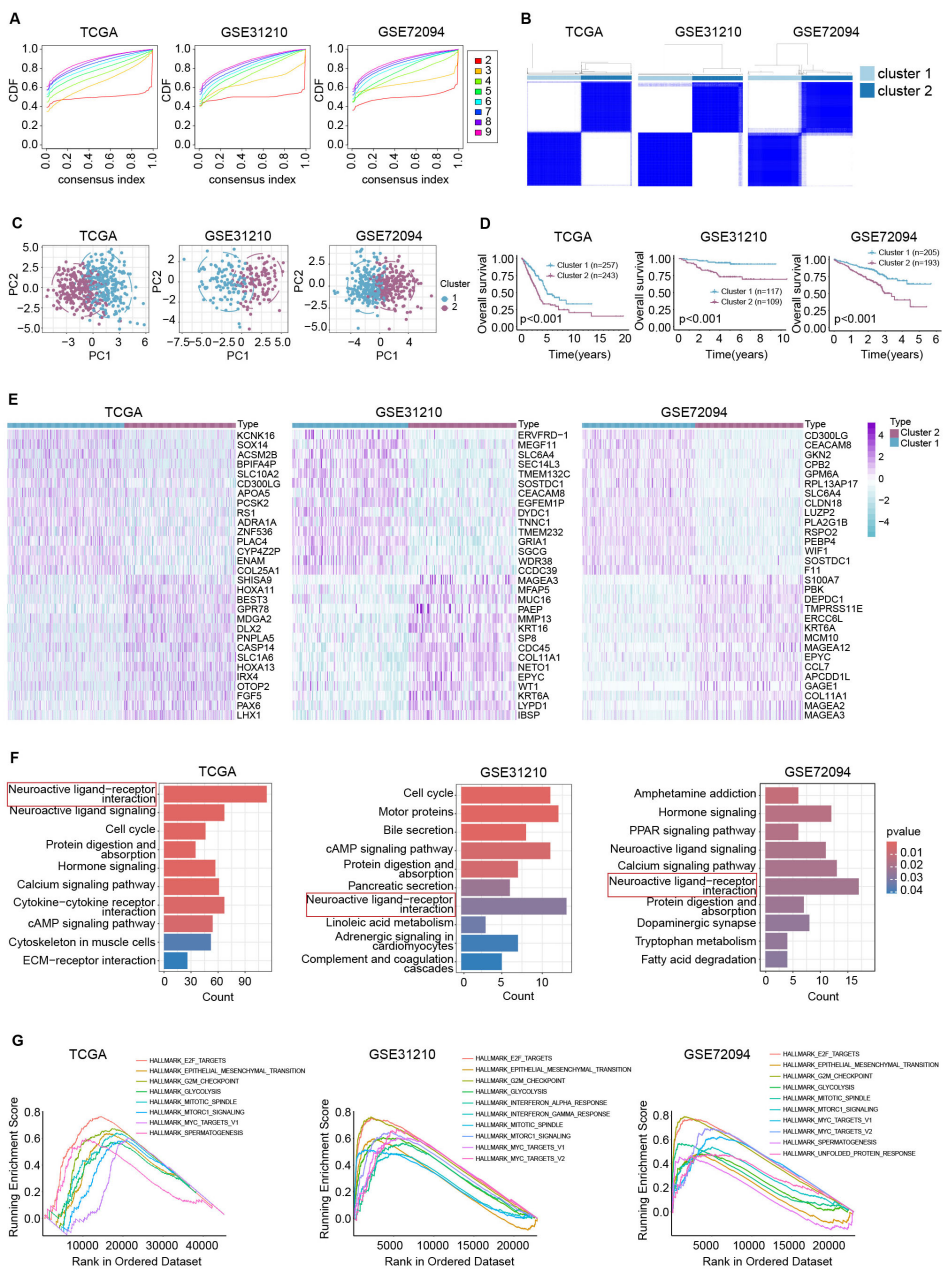
Classification of LUAD patients into two distinct clusters. **(A)** The consensus distribution for each k value was evaluated by empirical cumulative distribution function plot; **(B)** 17 hub DRGs divide LUAD patients into two molecular clusters (k=2); **(C)** PCAs were performed between cluster 1 and cluster 2; **(D)** survival analyses were conducted between cluster 1 and cluster 2; **(E)** heatmaps exhibited the top 15 variational genes in three cohorts; **(F)** KEGG analyses demonstrated the common involved signaling of differentially expressed genes between two clusters; **(G)** GSEAs showed the shared enriched signaling of differentially expressed genes.

To identify previously unrecognized depression-related subtypes, we performed unsupervised consensus clustering based on the aforementioned DRGs for LUAD patients. The optimal number of clusters (k=2) was determined, which revealed a significant divergence (**Figure 2A**), classifying LUAD patients into two distinct clusters (**Figures 2B–C**). Notably, patients in cluster 2 demonstrated worse overall survival compared with those in cluster 1 (**Figure 2D**). Subsequently, we conducted differential analysis in TCGA-LUAD, GSE31210, and GSE72094 (**Figure 2E**). KEGG analysis of the differentially expressed genes revealed significant enrichment in the “Neuroactive ligand−receptor interaction,” which has been reported to correlate with depression (18, 20) (**Figure 2F**). Additionally, GSEA indicated significant positive enrichment in several pathways, including “HALLMARK_E2F_TARGETS” (25–27), “HALLMARK_EPITHELIAL_MESENCHYMAL_TRANSITION” (28, 29), “HALLMARK_G2M_CHECKPOINT” (30), “HALLMARK_GLYCOLYSIS” (31–33), “HALLMARK_MTORC1_SIGNALING” (34–36), and “HALLMARK_MYC_TARGET” (37–39). These pathways may contribute to the pro-proliferative effects of depression (**Figure 2G**).

### Construction of depression-related signature for LUAD patients

**Figure 3:**
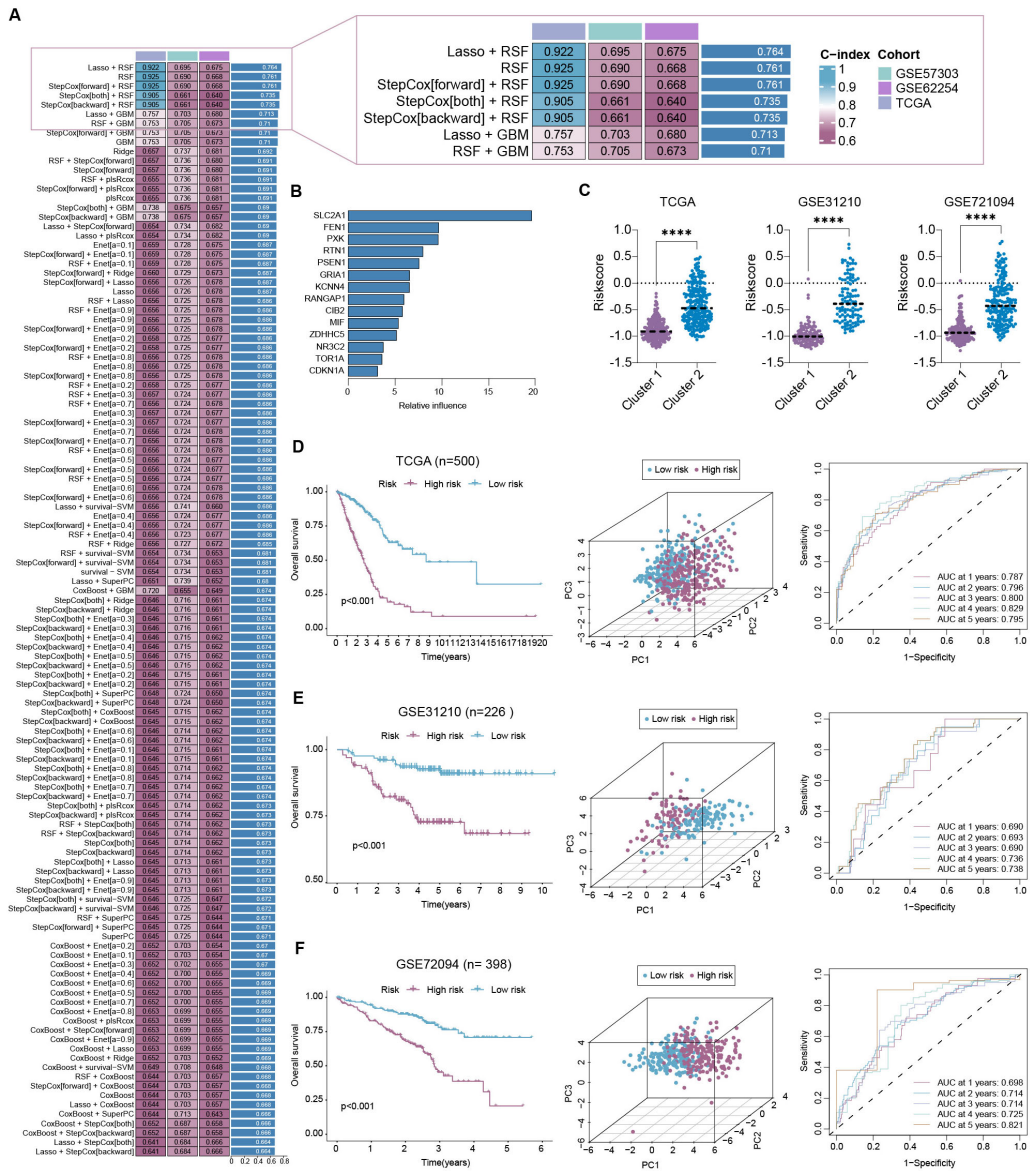
Machine learning-based construction of depression-related signature. **(A)** C-index values were calculated through 117 machine learning algorithms within TCGA-LUAD, GSE31210, and GSE72094; **(B)** relative influence of 14 hub DRGs in depression-related signature (DRS) were shown; **(C)** risk scores based on DRS were compared between cluster 1 and cluster 2 in three cohorts; **(D–F)** survival, PCA, and time-ROC analyses were conducted between high- and low-risk groups in TCGA-LUAD **(D)**, GSE31210 **(E)**, and GSE72094 **(F)**, respectively. ****P < 0.0001.

We performed 117 machine-learning algorithms to construct a depression-related signature (DRS) for LUAD patients based on the 17 hub DRGs identified earlier. TCGA-LUAD was used as the training set, whereas GSE31210 and GSE72094 served as the validation sets. Although several models, such as “LASSO + RSF,” “RSF,” “StepCox[forward] + RSF,” “StepCox[both] + RSF,” and “StepCox[backward] + RSF,” achieved high C-index values in TCGA-LUAD, their performance drastically declined in the validation sets, likely due to overfitting. Therefore, we selected the “LASSO + GBM” algorithm, which showed moderate performance across all three sets and yielded a model with an average C-index of 0.713 (**Figure 3A**). The constructed DRS, composed of 14 DRGs, was evaluated for the relative influence of each gene, including SLC2A1, FEN1, PXK, RTN1, PSEN1, GRIA1, KCNN4, RANGAP1, CIB2, MIF, ZDHHC5, NR3C2, TOR1A, and CDKN1A (**Figure 3B**). Risk scores were then calculated for each patient based on the relative influence of these 14 DRGs. Consistent with our expectations, we found that patients in cluster 2 had higher risk scores than those in cluster 1 across all three cohorts (**Figure 3C**).

Afterward, we divided LUAD patients into two groups based on the median risk score. Further analysis revealed that patients in the high-risk group had worse prognoses compared with those in the low-risk group, not only in the training set TCGA-LUAD (**Figure 3D**) but also in the validation sets GSE31210 (**Figure 3E**) and GSE72094 (**Figure 3F**). Additionally, principal component analysis (PCA) showed significant variation between the low- and high-risk groups across all cohorts (**Figures 3D–F**). Furthermore, time-dependent ROC curves demonstrated that the DRS performed stably for prognosis prediction, with high AUC values (**Figures 3D–F**).

### Evaluation of robustness of prognostic prediction of DRS

**Figure 4:**
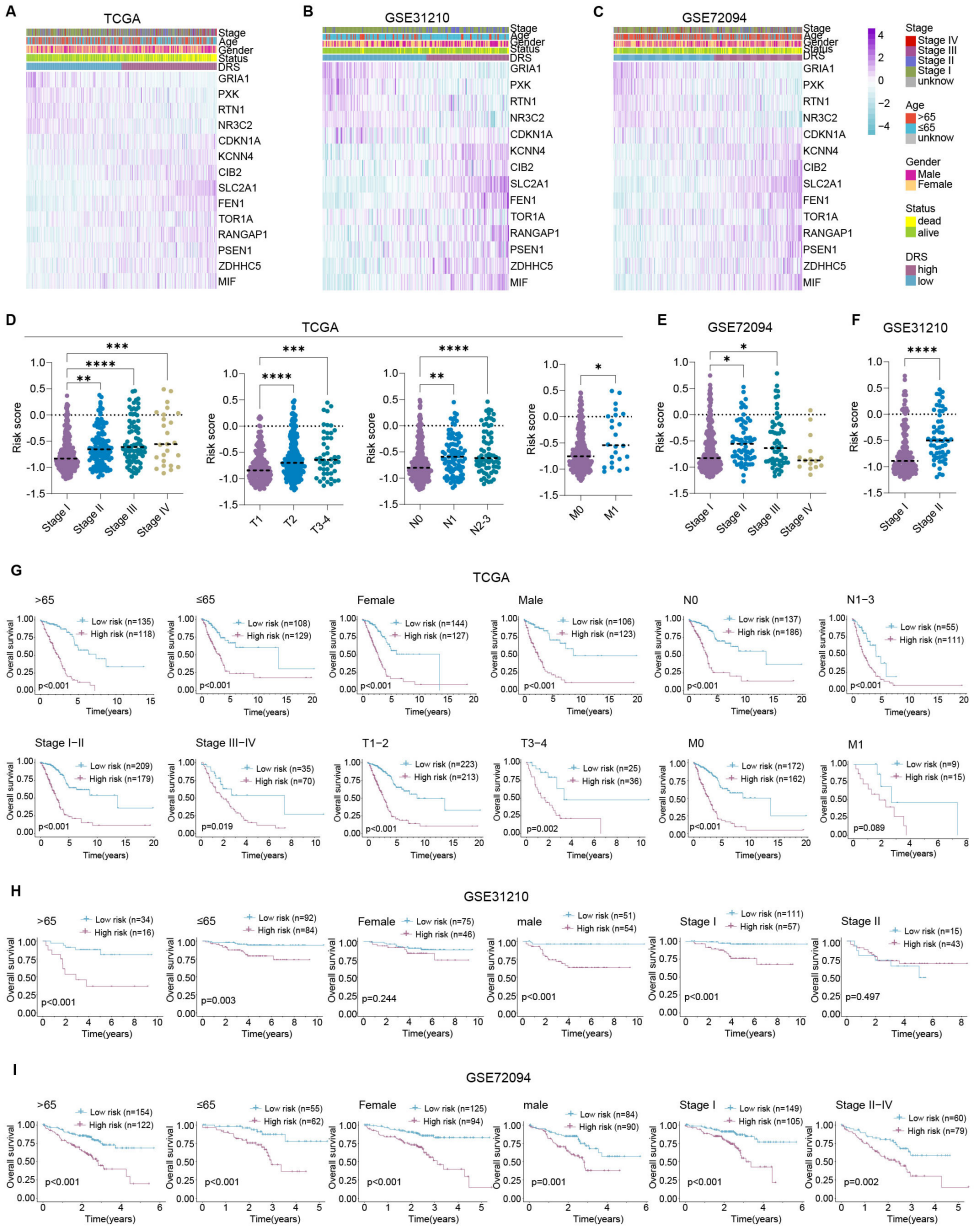
DRS acts as a robust prognostic indicator. **(A–C)** Heatmaps exhibit the expression profiles of DRS composing of 14 hub DRGs in TCGA-LUAD **(A)**, GSE31210 **(B)**, and GSE72094 **(C)**, respectively; **(D)** risk scores were contrasted amidst different clinical stages, T stages, N stages, and M stages in TCGA; **(E)** risk scores were compared among diverse clinical stages in GSE31210; **(F)** risk scores were calculated within different clinical stages in GSE72094; **(G)** comparative analyses of prognosis between diverse age, gender, clinical stage, T stage, N stage, and M stage subgroups in TCGA; **(H)** comparative analyses of prognosis between diverse age, gender, and clinical stage subgroups in GSE31210; **(I)** comparative analyses of prognosis between diverse age, gender, and clinical stage subgroups in GSE72094. *P < 0.05, **P < 0.01, ***P < 0.001, and ****P < 0.0001.

Heatmaps illustrated the transcriptional profiles of the 14 DRGs comprising the DRS across the three sets (**Figures 4A–C**). In the TCGA-LUAD set, patients with worse clinical characteristics exhibited higher risk scores, including those with advanced clinical stage, tumor invasiveness, lymphatic metastasis, and distant metastasis (**Figure 4D**). Consistently, patients in stage II or stage III had higher risk scores than those in stage I in both GSE31210 and GSE72094 (**Figures 4E, F**). Subsequent stratified survival analysis further demonstrated that LUAD patients in the high-risk group had poorer overall survival compared with those in the low-risk group across various subgroups, including those aged >65 years (p < 0.001), ≤65 years (p < 0.001), female (p < 0.001), male (p < 0.001), stages I-II (p = 0.044), stages III-IV (p = 0.019), T1-2 (p < 0.001), T3-4 (p = 0.002), N0 (p < 0.001), N1-N3 (p < 0.001), M0 (p < 0.001), and M1 (p = 0.089) in the TCGA-LUAD set (**Figure 4G**). Similarly, in the GSE31210 set, patients in the high-risk group exhibited worse outcomes compared with those in the low-risk group in the subgroups of >65 years (p < 0.001), ≤65 years (p = 0.003), female (p < 0.001), male (p < 0.001), and stage I (p < 0.001), but no significant differences were observed in the female (p = 0.244) or stage II (p = 0.497) subgroups (**Figure 4H**). In the GSE72094 set, high-risk patients had worse outcomes compared with low-risk patients in the >65 years (p < 0.001), ≤65 years (p < 0.001), female (p < 0.001), male (p = 0.001), stage I (p < 0.001), and stages II-IV (p = 0.002) subgroups (**Figure 4I**). These findings further highlight that the DRS serves as a robust prognostic indicator.

### Construction and assessment of DRS-based nomogram

**Figure 5:**
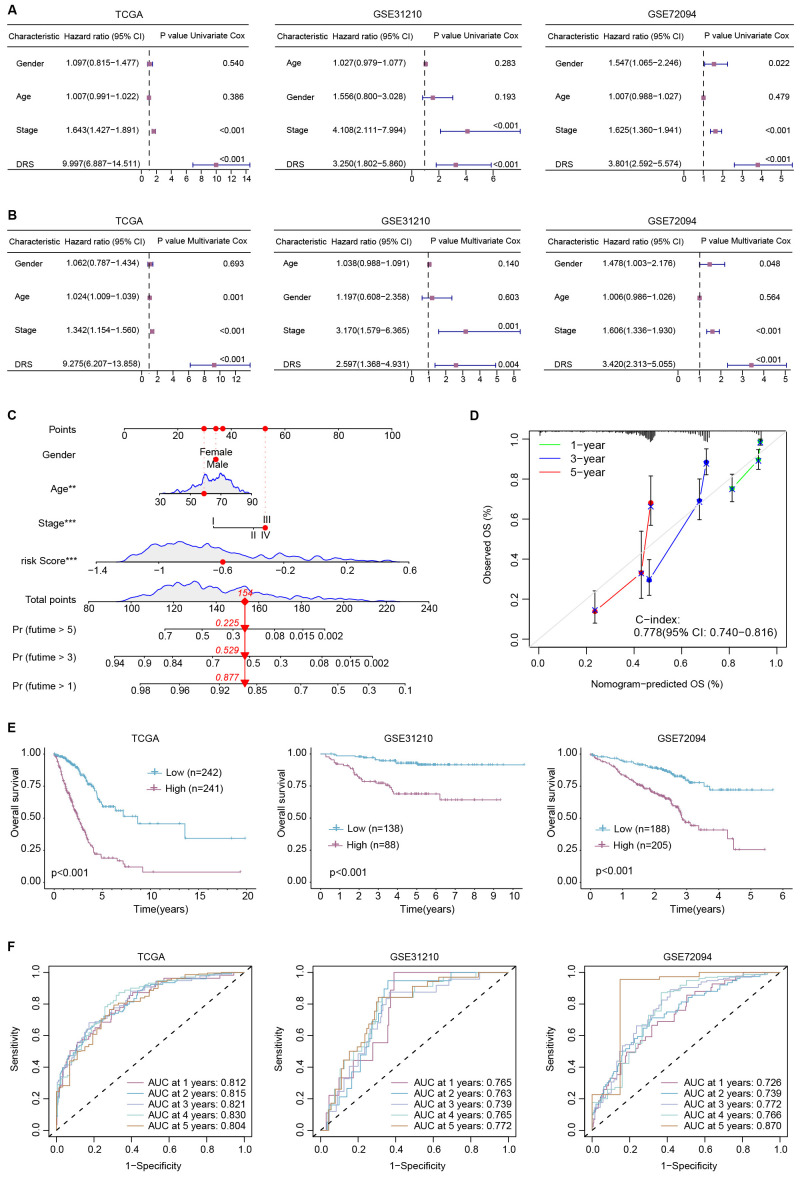
Integrated nomogram is developed for prognosis. **(A)** Univariate Cox regression was performed within age, gender, clinical stage, and DRS; **(B)** multivariate Cox regression was conducted within age, gender, clinical stage, and DRS; **(C)** the constructed nomogram is shown; **(D)** the C-index value was calculated with calibration curves; **(E)** patients in the high nomogram group exhibited worse overall survival in TCGA-LUAD, GSE31210, and GSE72094; **(F)** time-ROC analyses showcase the robust and remarkable predictive efficacy of the constructed nomogram within three cohorts.

Both univariate Cox regression (**Figure 5A**) and multivariate Cox regression (**Figure 5B**) indicated that DRS is an independent prognostic predictor for LUAD. Based on this, we constructed a nomogram integrating age, gender, clinical stage, and DRS for overall survival prediction in LUAD (**Figure 5C**). The constructed nomogram achieved a C-index of 0.778, and calibration curves confirmed the accuracy of survival probability estimations at 1-, 3- and 5-year intervals (**Figure 5D**). Moreover, we observed a significant difference in survival probability between high- and low-nomogram patients (**Figure 5E**). Time-dependent ROC curves demonstrated that the AUCs for 1-, 2-, 3-, 4-, and 5-year survival predictions were 0.812, 0.815, 0.821, 0.830, and 0.804 in the TCGA-LUAD set, 0.765, 0.763, 0.739, 0.765, and 0.772 in GSE31210, and 0.726, 0.739, 0.772, 0.766 and 0.870 in GSE72094 (**Figure 5F**).

### Immune landscape of groups stratified by DRS

**Figure 6:**
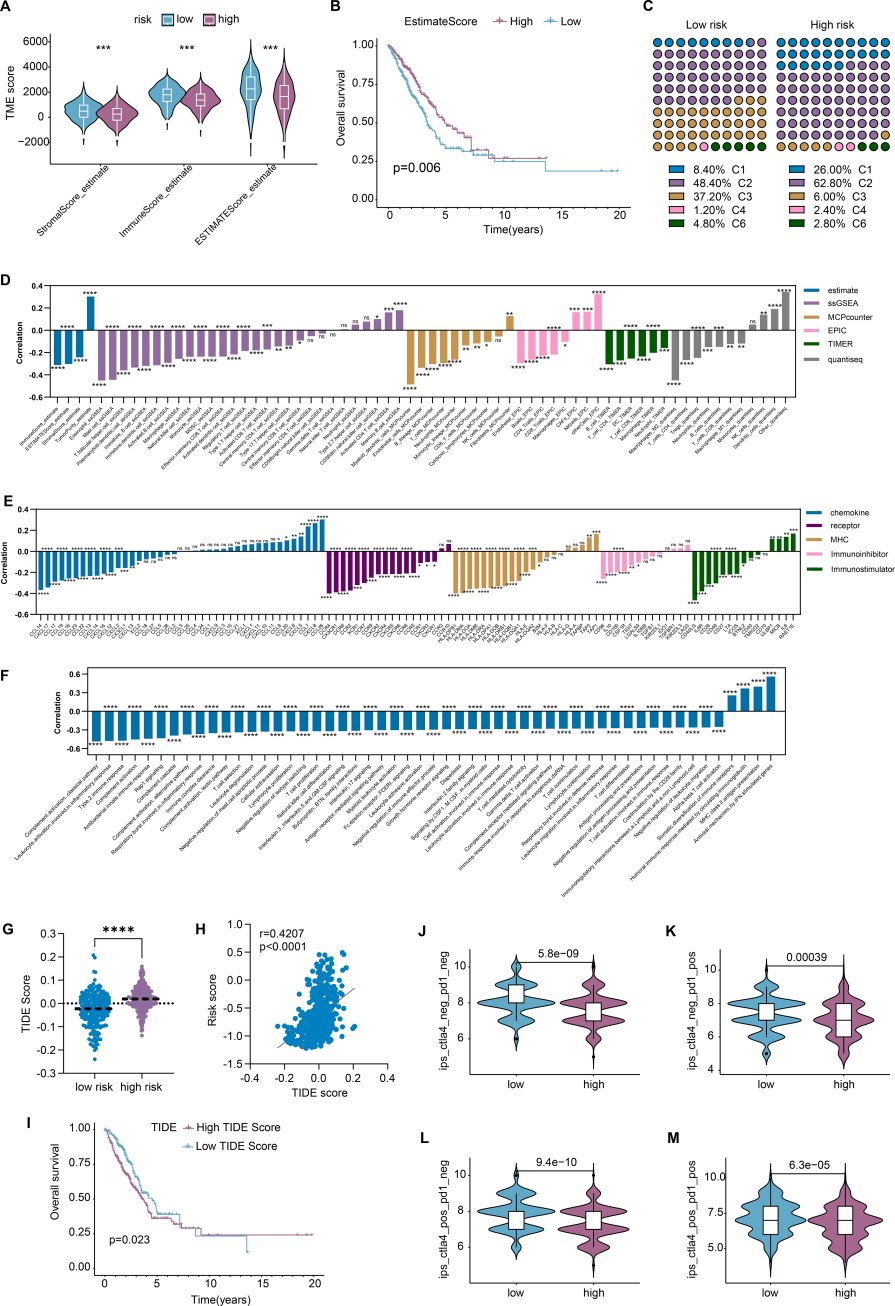
Unveil the involvement of DRS in tumor immune microenvironment (TME). **(A)** Patients in the high-risk group exhibited lower TME scores with the ESTIMATE algorithm; **(B)** low ESTIMATE score indicated worse survival probability; **(C)** patients in the high-risk group were more likely to stratify into C1 and C2 but C3, C4, or C6 immune subgroups than that in the low-risk group; **(D)** a negative correlation was observed between risk score and the infiltration of most immune cells; **(E)** risk score was negatively correlated with the majority of TME modulators; **(F)** a negative relationship was detected between risk score and a considerable number of immune pathways; **(G)** high-risk group patients showed higher TIDE scores, indicating weaker responsiveness to immunotherapy; **(H)** risk score was positively correlated with TIDE score; **(I)** high TIDE score indicated worse outcome; **(J–M)** high-risk group patients gained lower IPS in all four subtypes, indicating worse responses to corresponding therapy. *P < 0.05, **P < 0.01, ***P < 0.001, and ****P < 0.0001.

We first evaluated the tumor microenvironment using the ESTIMATE algorithm. The results showed that patients in the high-risk group had lower immune scores, stromal scores, and ESTIMATE scores compared with those in the low-risk group (**Figure 6A**), which correlated with worse clinical outcomes (**Figure 6B**). To further investigate, we examined the immune subtypes in different groups and found that patients in the high-risk group were more likely to be classified into C1 and C2 subgroups but less likely to be classified into C3, C4, or C6, compared with those in the low-risk group, potentially indicating a higher proliferation rate (**Figure 6C**). Additionally, six algorithms were used to detect the immune cell infiltration. The results indicated that risk score was negatively correlated with most immune cells, except for tumor cells, fibroblasts, and a few others (**Figure 6D**). Negative correlations were also observed between the risk score and several immune checkpoints (**Figure 6E**). Moreover, single-sample gene set enrichment analysis (ssGSEA) with the IMMUPORT database revealed that risk score was negatively correlated with most immune pathways, such as “complement.activation,” “Leukocyte.activation,” “T.cell.proliferation,” and others (**Figure 6F**).

Based on these findings, we noted that patients in the high-risk group exhibited weaker immune characteristics, potentially indicating a lower therapeutic response to immune interventions. Consequently, we calculated the TIDE scores and found that patients in the high-risk group had higher TIDE scores than those in the low-risk group (**Figure 6G**), suggesting weaker responsiveness to immunotherapy. Moreover, a significant positive correlation was observed between the risk score and the TIDE score (**Figure 6H**), which was closely related to the overall survival (**Figure 6I**). Consistently, we calculated the immunophenoscore (IPS) using The Cancer Immunome Atlas (TCIA) and found that patients in the high-risk group had lower IPS values compared with those in the low-risk group, indicating poorer responses to anti-cytotoxic T lymphocyte antigen-4 (CTLA-4) and anti-programmed cell death protein 1 (anti-PD-1) therapies (**Figures 6J–M**).

### Sensitivity to chemotherapy and targeted therapy classified by DRS

**Figure 7:**
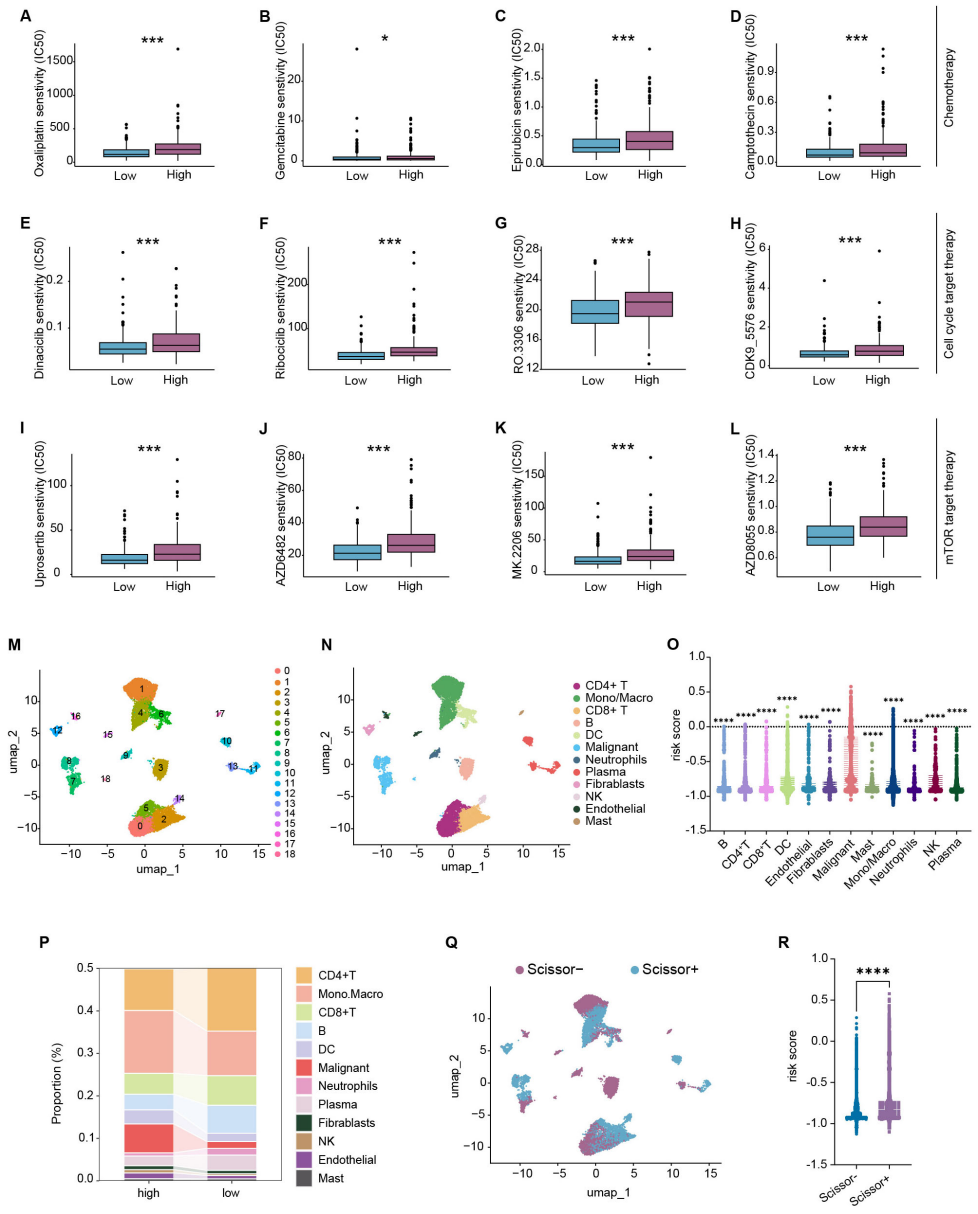
DRS influences therapeutic responses and acts as a risk indicator in cellular level. **(A–D)** Patients in the high-risk group exhibited higher IC50 to chemotherapy, such as oxaliplatin **(A)**, gemcitabine **(B)**, epirubicin **(C)**, and camptothecin **(D)**; **(E–H)** patients in the high-risk group gained higher IC50 to targeted therapy toward the cell cycle, such as dinaciclib **(E)**, ribociclib **(F)**, RO.3306 **(G)**, and CDK9_5576 **(H)**; **(I–L)** patients in the high-risk group obtained higher IC50 to molecular therapy targeting mTOR signaling, such as uprosertib **(I)**, AZD6482 **(J)**, MK.2206 **(K)**, and AZD8055 **(L)**; **(M)** Umap visualization of different clusters; **(N)** Umap visualization of different cell types; **(O)** dot plot showing the risk scores of different cell types; **(P)** bar plot showing the proportion of different cell types in the high- and low-risk groups; **(Q)** Umap visualization of Scissor+ and Scissor− cells; **(R)** dot plot showing the risk scores between Scissor+ and Scissor− cells. *P < 0.05, ***P < 0.001, and ****P < 0.0001.

To gain deeper insights, we further evaluate the response of high- and low-risk groups to chemotherapy and specific targeted therapies using the oncoPredict algorithm. The results showed that patients in the high-risk group had a higher half maximal inhibitory concentration (IC50) for traditional chemotherapy such as oxaliplatin (**Figure 7A**), Gemcitabine (**Figure 7B**), epirubicin (**Figure 7C**), and camptothecin (**Figure 7D**), compared with those in the low-risk group. In our previous study, we demonstrated that depression-related genes are closely associated with pro-proliferation pathways, such as “HALLMARK_E2F_TARGETS”, “HALLMARK_G2M_CHECKPOINT”, “HALLMARK_GLYCOLYSIS”, and “HALLMARK_MYC_TARGET”, as well as pro-migration pathways such as “HALLMARK_EPITHELIAL_MESENCHYMAL_TRANSITION” and “HALLMARK_MTORC1_SIGNALING,” which contribute to malignant behaviors in lung cancer. These findings are consistent with previous studies. Therefore, we explored therapeutic responses to drug targeting proliferation-related “CDH family” and migration-related “mTOR signaling”. The results revealed that patients in the high risk group were less sensitive to “CDH family”-targeted therapies, such as “Dinaciclib” (**Figure 7E**), “Ribociclib” (**Figure 7F**), “RO.3306” (**Figure 7G**), and “CDK9_5576” (**Figure 7H**). Similarly, high-risk patients had higher IC50 values for “mTOR signaling” targeted drugs, such as “Uprosertib” (**Figure 7I**), “AZD6482” (**Figure 7J**), “MK.2206” (**Figure 7K**), and “AZD8055” (**Figure 7L**), indicating poorer therapeutic responses to these treatments. Additionally, negative correlations were observed between risk scores and IC50 values (**Supplementary Figure 1A**). We also performed Connectivity Map (CMap) analysis on the differentially expressed genes between the high- and low-risk groups. The results suggested that HDAC inhibitors “Vorinostat,” “ISOX,” and “THM-I-94” as well as the LRRK2 inhibitor “XMD-1150” were optimal for patients in the high-risk group (**Supplementary Figure 1B**).

### Processing of single-cell RNA sequencing and Scissor algorithm

In the three independent bulk datasets, the DRS acts as a critical risk indicator for LUAD patients. This led us to investigate whether DRS plays a similar role at the cellular level. We first processed single-cell RNA sequencing data from GSE127465 using the Seurat package. Quality control and cell identification were performed as indicated (**Supplementary Figures 1C–H**). We identified 12 distinct cell populations (**Figures 7M, N**, **Supplementary Figure 1E**) and calculated the risk score for each cell. The results showed that malignant cells had highest risk scores among all other cell types (**Figure 7O**). Additionally, the high-risk group had a higher proportion of malignant cells (**Figure 7P**). Next, we used TCGA-LUAD as a reference to bridge depression phenotypes to the cellular level. Ultimately, we identified 5,963 Scissor+ (high-risk) cells and 7,099 Scissor− (low-risk) cells (**Figure 7Q**). We confirmed that Scissor+ cells exhibited significantly higher risk scores than Scissor− cells (**Figure 7R**).

### Validation of DRS with a depression-like mouse model

**Figure 8:**
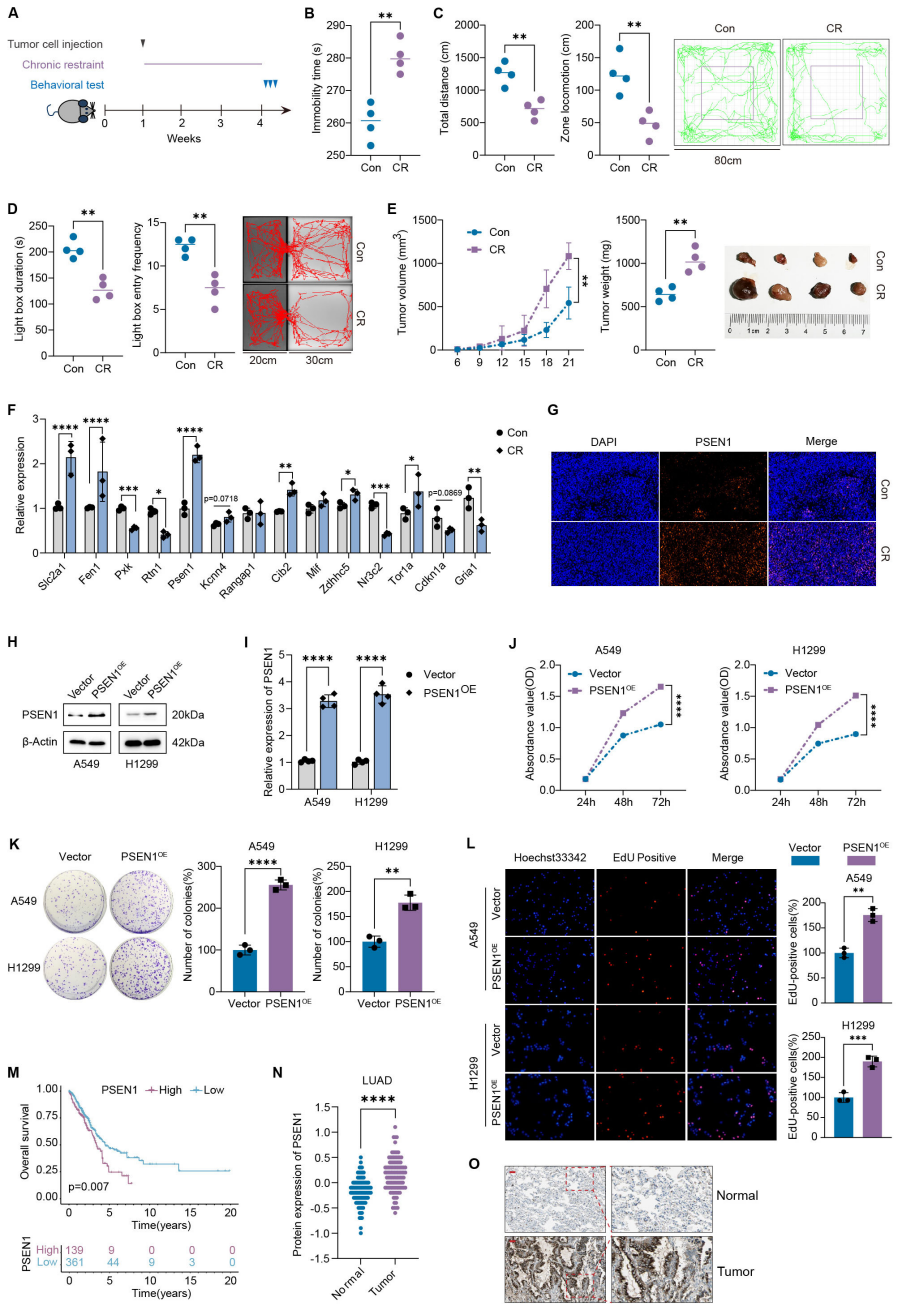
PSEN1 induced by depression promotes LUAD proliferation. **(A)** Process of the animal experiment; **(B)** immobility times in the forced swimming test (FST) were compared between Con and CR mice (n=4); **(C)** total locomotion and central zone activity in OFT were compared between Con and CR mice (n=4); **(D)** total duration and migration to light box in LDBT were compared between Con and CR mice (n=4); **(E)** the LLC tumor growth curves and the end-point tumor weights and sizes were represented (n=4); **(F)** the mRNA levels of 14 DRGs in tumor tissues were tested by qRT-PCR (n=3); **(G)** immunofluorescence detection of PSEN1 protein expression in tumor tissues; **(H)** Validation of the overexpression of PSEN1 in A549 and H1299 cells by western blot; **(I)** validation of the overexpression of PSEN1 in A549 and H1299 cells by qRT-PCR; **(J–L)** determine the effect of PSEN1 overexpression on the proliferation of A549 and H1299 cells through CCK-8 **(J)**, colony formation assay **(K)**, and EdU **(L)**; **(M)** survival curve of LUAD patients with low or high expression of PSEN1; **(N)** protein levels of PSEN1 in normal tissue and LUAD in CPATC database; **(O)** representative images ofimmunohistochemistry of PSEN1 in normal tissue and LUAD in HPA database. Data are represented as the mean ± standard error of the mean (SEM). *P < 0.05, **P < 0.01, ***P < 0.001, and ****P < 0.0001 by an unpaired Student’s t-test.

To investigate the potential impact of depression on LUAD development, we used chronic restraint stress (CR) models to construct a depression-like LUAD mouse model (**Figure 8A**). Repeated exposure to restraint stress resulted in prolonged immobility time (**Figure 8B**), diminished locomotion, and exploration in the central area during the open-field test (**Figure 8C**), and declined exploration of the light compartment in the light–dark box test (**Figure 8D**). The significant upregulation of ACTH and COR in serum further confirmed the stress state in the mice (**Supplementary Figure 2D**). We next assessed the effect of CR on tumor growth in mice by measuring the volume and weight of subcutaneous tumors. CR exposure led to increased tumor proliferation (tumor size in mm^3^) compared with non-stressed controls (Con) (**Figure 8E**). The depression-related signature (DRS) composed of 14 DRGs, including SLC2A1, FEN1, PXK, RTN1, PSEN1, GRIA1, KCNN4, RANGAP1, CIB2, MIF, ZDHHC5, NR3C2, TOR1A, and CDKN1A (**Figure 3B**), was validated in tumor tissues. The results showed that the elevated expression of SLC2A1, FEN1, and PSEN1 was most prominent in CR mice compared with non-depressed mice (**Figure 8F**). Immunofluorescence analysis also showed increased expression of PSEN1 in the tumor tissues of CR mice (**Figure 8G**).

### PSEN1 facilitates LUAD cell proliferation induced by depression

When A549 cells were treated with COR, the expression level of PSEN1 increased (**Supplementary Figure 2A**), accompanied by a decrease in cell apoptosis (**Supplementary Figure 2B**) and an acceleration in cell proliferation (**Supplementary Figure 2C**). To confirm the effect of PSEN1 on lung cancer, we first established PSEN1 overexpression in A549 and H1299 cells, which was validated by western blotting (**Figure 8H**) and qRT-PCR (**Figure 8I**). *In vitro*, cell proliferation was enhanced following PSEN1 overexpression in both A549 and H1299, as determined by CCK8 (**Figure 8J**), colony formation (**Figure 8K**), and EdU assays (**Figure 8L**). Protein quantification indicated that PSEN1 was significantly upregulated in LUAD tissues compared with normal tissues (**Figure 8N**), a finding confirmed by immunohistochemistry (IHC) as well (**Figure 8O**). The high expression of PSEN1 correlated with poor overall survival in LUAD patients (**Figure 8M**). Next, we explored the downstream signaling pathways through which PSEN1 promotes LUAD proliferation. To investigate this, we performed enrichment analyses. Both Kyoto Encyclopedia of Genes and Genomes (KEGG, **Supplementary Figure 3A**) and Gene Set Enrichment Analysis (GSEA, **Supplementary Figure 3B**) based on TCGA-LUAD indicated an association between PSEN1 and ErbB signaling using the BEST database (https://rookieutopia.hiplot.com.cn/app_direct/BEST/). Consistently, western blotting confirmed that overexpression of PSEN1 promoted phosphorylation of ERBB1 and activation of the PI3K-AKT signaling pathway, which could be inhibited by PI3K inhibitor LY294002 (**Supplementary Figure 2F**). Accordingly, *in vitro* experiments showed that the pro-proliferative effect of PSEN1 overexpression was attenuated by LY294002 (**Supplementary Figure 2E**). Further *in vivo* experiments corroborated the tumor-promoting role of PSEN1 and demonstrated that the PI3K inhibitor Alpelisib could reverse this effect (**Supplementary Figure 2G**). Collectively, these results suggest that stress-induced PSEN1 may facilitate LUAD progression by activating the ERBB1-PI3K-AKT signaling pathway, a well-established driver of lung adenocarcinoma proliferation.

## Discussion

Lung adenocarcinoma (LUAD) remains a significant public health issue worldwide due to its high incidence and mortality rates (2, 40). The progression and prognosis of LUAD are influenced by various factors, such as tumor biology (41, 42), clinical staging (43–45), and host factors (46). Among these, emerging studies have shown that depression is closely correlated with the occurrence and prognosis of LUAD (10). Depression, also known as major depressive disorder, is a common but serious mood disorder characterized by persistent sadness, loss of interest, and impaired daily functioning. It affects over 280 million people globally, making it a leading cause of disability worldwide (47). Therefore, further investigation into how depression affects LUAD is critical, as it could help identify appropriate treatments or targeted therapies for LUAD patients with depression.

In our recent study, we first identified 692 depression-related genes and found that these genes were enriched in biological process (BP) such as “cognition” and “dopamine metabolic process,” as well as KEGG pathways like “Neuroactive ligand signaling” and “Neuroactive ligand−receptor interaction,” which is consistent with previous studies. We then performed unsupervised consensus clustering, classifying patients into two distinct subtypes with differing prognoses and characteristics, as depicted in **Figure 2**. We subsequently constructed a depression-related signature (DRS) using 17 overlapping hub depression-related genes (DRGs) and 117 machine-learning algorithms. The LASSO + GBM algorithm performed robustly across TCGA-LUAD, GSE31210, and GSE72094, yielding an average C-index of 0.713, which is shown in **Figure 3C**. Furthermore, we uncovered that DRS was correlated with clinical outcomes as well as clinicopathologic features such as clinical stage, tumor invasiveness, lymphatic metastasis, and distant metastasis. Moreover, stratified survival analysis, univariate cox regression, and multivariate cox regression confirmed that DRS serves as an independent prognostic indicator. Additionally, a nomogram constructed with DRS, age, gender, and clinical stage demonstrated commendable performance.

Our DRS is composed of 14 hub DRGs with varying relative influences, including SLC2A1 (19.55), FEN1 (9.65), PXK (9.59), RTN1 (7.96), PSEN1 (7.55), GRIA1 (6.52), KCNN4 (6.51), RANGAP1 (5.94), CIB2 (5.77), MIF (5.34), ZDHHC5 (5.14), NR3C2 (3.74), TOR1A (3.60), and CDKN1A (3.13). Consistent with our findings, Zhou Z reported that SLC2A1, also known as GLUT1, promotes proliferation by binding to and stabilizing phosphorylated EGFR in LUAD (48). Similarly, suppression of FEN1 restricts tumor progression and induces apoptosis by inhibiting DNA replication and accumulating DNA damage in LUAD (49). Furthermore, RTN1 expression is decreased and associated with worse outcomes in LUAD (50). However, the role of PSEN1 in LUAD progression remains unclear. Therefore, we overexpressed PSEN1 in A549 and H1299 cell lines and found that PSEN1 overexpression promoted cell proliferation in LUAD, as confirmed by CCK-8, colony formation, and EdU assays. Additionally, depression-like mouse models were constructed to investigate the involvement of these DRGs in LUAD development. The results showed varying degrees of expression changes in the 14 DRGs, with SLC2A1, FEN1, and PSEN1 showing the most significant and dominant alterations. Consistently, former studies indicated that PSEN1 is involved in the occurrence and progression of depression (51, 52). Thus, PSEN1 may mediate depression-induced LUAD progression.

The tumor microenvironment is known to have a significant impact on tumor progression and clinical outcomes (53, 54). Using multiple algorithms to calculate immune cell infiltration, we found that most immune cells, except tumor cells and fibroblasts, were significantly negatively correlated with the risk score calculated by DRS, as depicted in **Figure 6**. Additionally, the risk score was negatively correlated with the majority of immune checkpoints and immune pathways, indicating that LUAD patients in the high-risk group exhibited weaker “immune hot” features compared with those in the low-risk group. Previous studies have shown that depression diminishes the effectiveness of various therapeutic responses, including chemotherapy (55–58), radiotherapy (59–61), and immunotherapy (62–65). Therefore, we calculated TIDE scores and immunophenoscore (IPS) and found that patients in the high-risk group had lower therapeutic responses to immune interventions, further confirming the impact of depression on the immunotherapy. To explore this further, we discovered that patients in the high-risk group had high IC50 values for traditional chemotherapy and specific targeted therapy, indicating worse therapeutic efficacy and clinical outcomes. To identify potentially effective targeted drugs for high-risk patients, we conducted CMap analysis and found that HDAC inhibitors “Vorinostat,” “ISOX,” and “THM-I-94,” and the LRRK2 inhibitor “XMD-1150” were optimal for high-risk group patients. In summary, depression generally leads to poorer therapeutic responses, and further studies are needed to clarify how depression affects therapeutic sensitivity. To further validate the effect of DRS at the cellular level, we performed Scissor algorithm analysis with single-cell RNA sequencing and found that Scissor+ cells had higher risk scores than Scissor− cells.

In conclusion, DRS can serve as an independent prognostic indicator for LUAD patients, and PSEN1 may contribute to depression-induced LUAD progression. More importantly, investigating the molecular features based on DRS could enhance individualized treatment and clinical decision-making for LUAD patients.

## Data Availability

The original contributions presented in the study are included in the article/**Supplementary Material**. Further inquiries can be directed to the corresponding author.
