# Supplementary material for: Novel prognostic signature unveils PSEN1 contributes to depression-induced lung adenocarcinoma progression
Source: Front Immunol. 2026 Jan 29;17:1681306. doi: 10.3389/fimmu.2026.1681306 (PMC12893990; doi:10.3389/fimmu.2026.1681306)
Supplement: Supplementary file 1 [file DataSheet1.pdf]

Supplementary table 1. qRT-PCR Primer Sequence

Gene Name	Forward Primer	Reverse Primer
Gapdh	CTGCCCAGAACATCATCC	CTCAGATGCCTGCTTCAC
Slc2a1	TCAAACATGGAACCACCGCTA	AAGAGGCCGACAGAGAAGGAA
Fen1	ACCAGTTCCTGATTGCTGTTC	TCATGCGGATGGTACGGTAGA
Pxk	CATCCGGGTGCAAAGAGGAAT	CTCACGGTCCATGTTTCCAAT
Rtn1	CCGGACCCTCGTTACCAGA	TCAGCTAGGATCTTGTTCACCT
Psen1	TGCACCTTTGTCCTACTTCCA	GCTCAGGGTTGTCAAGTCTCTG
Gria1	CAAGTTTTCCCGTTGACACATC	CGGCTGTATCCAAGACTCTCTG
Kcnn4	TTCAACAAGGCGGAGAAACAC	TCTTGCGCTGATGTCTGCG
Rangap1	CACTAGGGGAGGGACTCATCA	CACTCCATCGGGTCCAAATG
Cib2	TCTGTGCTCTGCGAATCAGC	TGGCGATCATGTCCTCAAAGT
Mif	GAGGGGTTTCTGTCGGAGC	GTTCGTGCCGCTAAAAGTCA
Zdhhc5	CAAACCCAGCAAGTATGTACCG	CTGGACACGTAAAGGCAAAGA
Nr3c2	GAAAGGCGCTGGAGTCAAGT	CCATGTAGCTGTTCTCATTGGT
Tor1a	CAACCCGAAGCCCAAGAAG	GTTCAGTCCGCCCTCGTAAAT
Cdkn1a	CCTGGTGATGTCCGACCTG	CCATGAGCGCATCGCAATC
GAPDH	ACAACTTTGGTATCGTGGAAGG	GCCATCACGCCACAGTTTC
PSEN1	GACGACCCCAGGGTAACTC	ACTGACTTAATGGTAGCCACGA
