# Supplementary material for: Novel prognostic signature unveils PSEN1 contributes to depression-induced lung adenocarcinoma progression
Source: Front Immunol. 2026 Jan 29;17:1681306. doi: 10.3389/fimmu.2026.1681306 (PMC12893990; doi:10.3389/fimmu.2026.1681306)
Supplement: Supplementary file 2 [file DataSheet2.pdf]

Novel prognostic signature unveils PSEN1 contributes to depression-induced lung adenocarcinoma progression

Qiaoqi Zheng[1, 2], Ji Zhuoga[1, 2], Congcong Li[1, 2, 3], Wenjing Chen[1, 2, 3], Maimaitituson Yalikun[1, 2, 3], Peng Fu[3], Zaiquan Dong[3,4*], Jingcheng Dong[1, 2, 3*]

1. Department of Integrative Medicine, Huashan Hospital, Fudan University, Shanghai, China
2. Institute of Integrative Medicine, Fudan University, Shanghai, China
3. The Mental Rehabilitation Centers, Karamay Municipal People's Hospital, Karamay, China
4. Mental Health Center, West China Hospital, Sichuan University, Chengdu, China

***Corresponding author:**

Jingcheng Dong Prof.

Email: jcdong2004@126.com

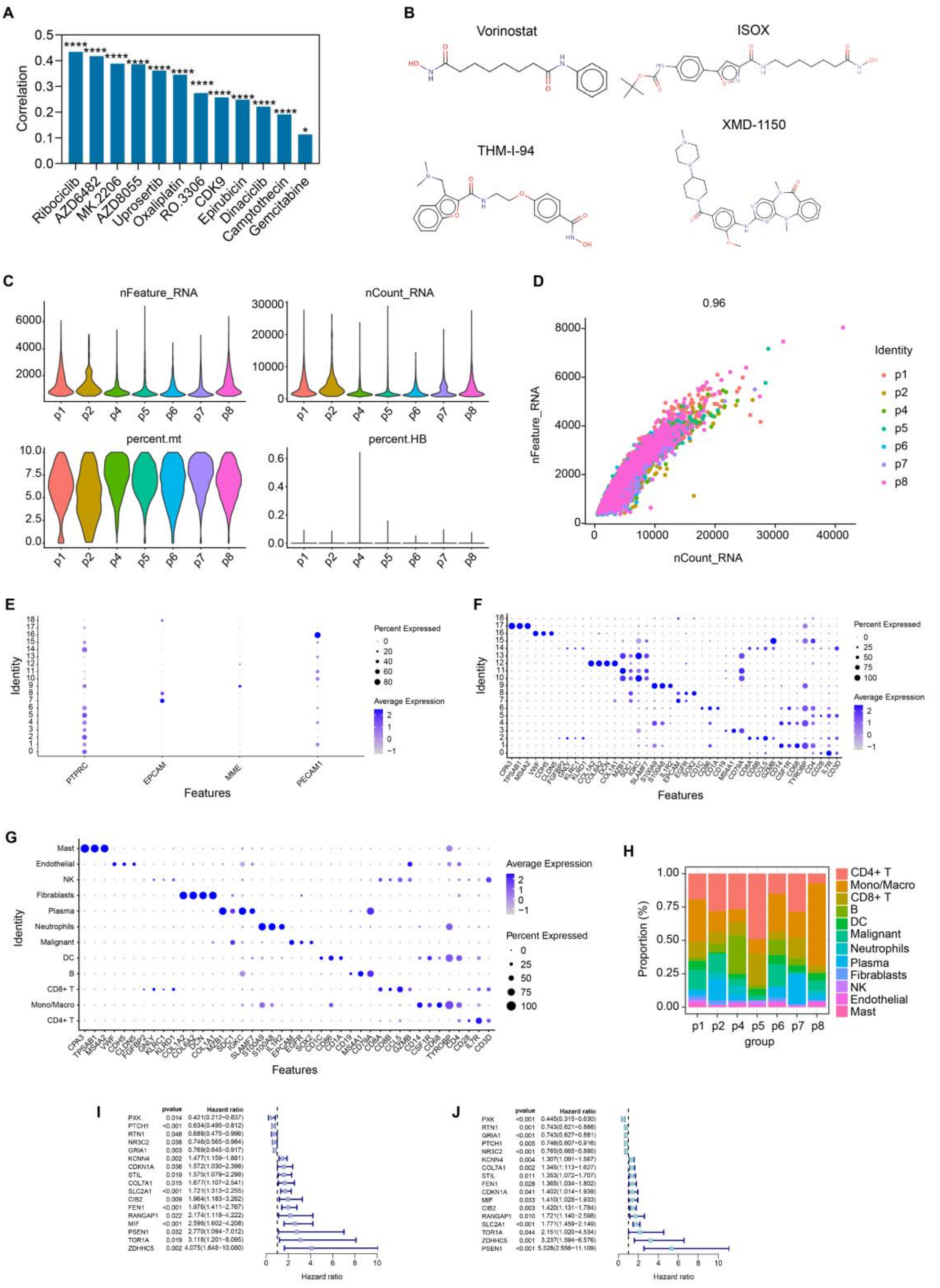

Supplementary Figure. 1 DRS affects therapeutic responses and works as a risk indicator in cellular level

(A) A positive correlation was observed between risk score and IC50 of chemotherapy or specific targeted therapy; **(B)** Ideal molecular therapies were selected for patients in high-risk group; **(C)** Quality check of single-cell RNA sequencing of GSE127465; **(D)**

The correlation of the number of genes (nFeature) and the sequence count per cell (nCount) was shown; **(E&F)** Dot plot of marker genes of different clusters; **(G)** Dot plot of marker genes of different cell types; **(H)** Proportion of different cell types in diverse samples; **(I)** Univariate Cox regression indicated the hazard ratios of 17 hub DRGs in GSE31210; **(J)** Univariate Cox regression indicated the hazard ratios of 17 hub DRGs in GSE72095.

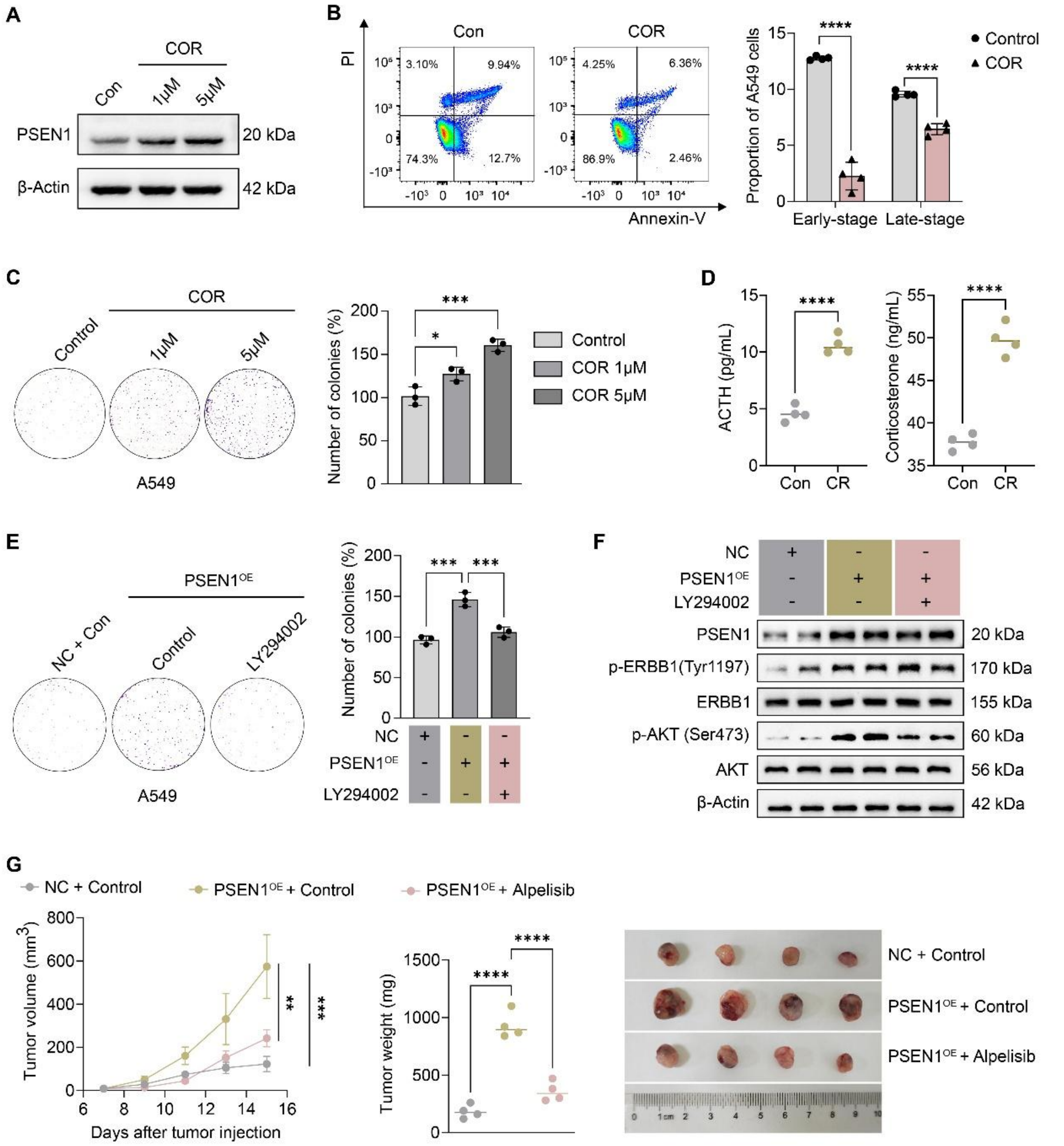

Supplementary Figure. 2 PSEN1 promotes LUAD progression by regulating the ERBB1-PI3K-AKT pathway

(A) After COR treatment, the protein level of PSEN1 was tested by Western blot; **(B)**

Detection of the effect of COR on apoptosis of A549 cells by Flow cytometry; **(C)** Colony formation assay for detecting the effect of COR on A549 cell proliferation; **(D)** The concentration of adrenocorticotropic hormone (ACTH) and corticosterone (COR) in serum were tested by ELISA between the control (Con) and chronic restraint (CR) mice (n = 4). *$p < 0.05$, ***$p < 0.001$ and ****$p < 0.0001$ by an unpaired Student's t-test; **(E)** Colony formation assay was used to detect the effect of PSEN1 overexpression and PI3K inhibitor LY294002 on A549 cell proliferation; **(F)** The activation of the ERBB1-PI3K-AKT signaling pathway of A549 cells after PSEN1 overexpression and LY294002 treatment was tested by Western blot; **(G)** The A549 tumor growth curves, the end-point tumor weights and sizes of mice were represented (n=4). In vivo validation of the effects of PSEN1 overexpression and PI3K inhibitor Alpelisib on the progression of LUAD. **$p < 0.01$, ***$p < 0.001$ and ****$p < 0.0001$ by one-way analysis of variance (ANOVA).

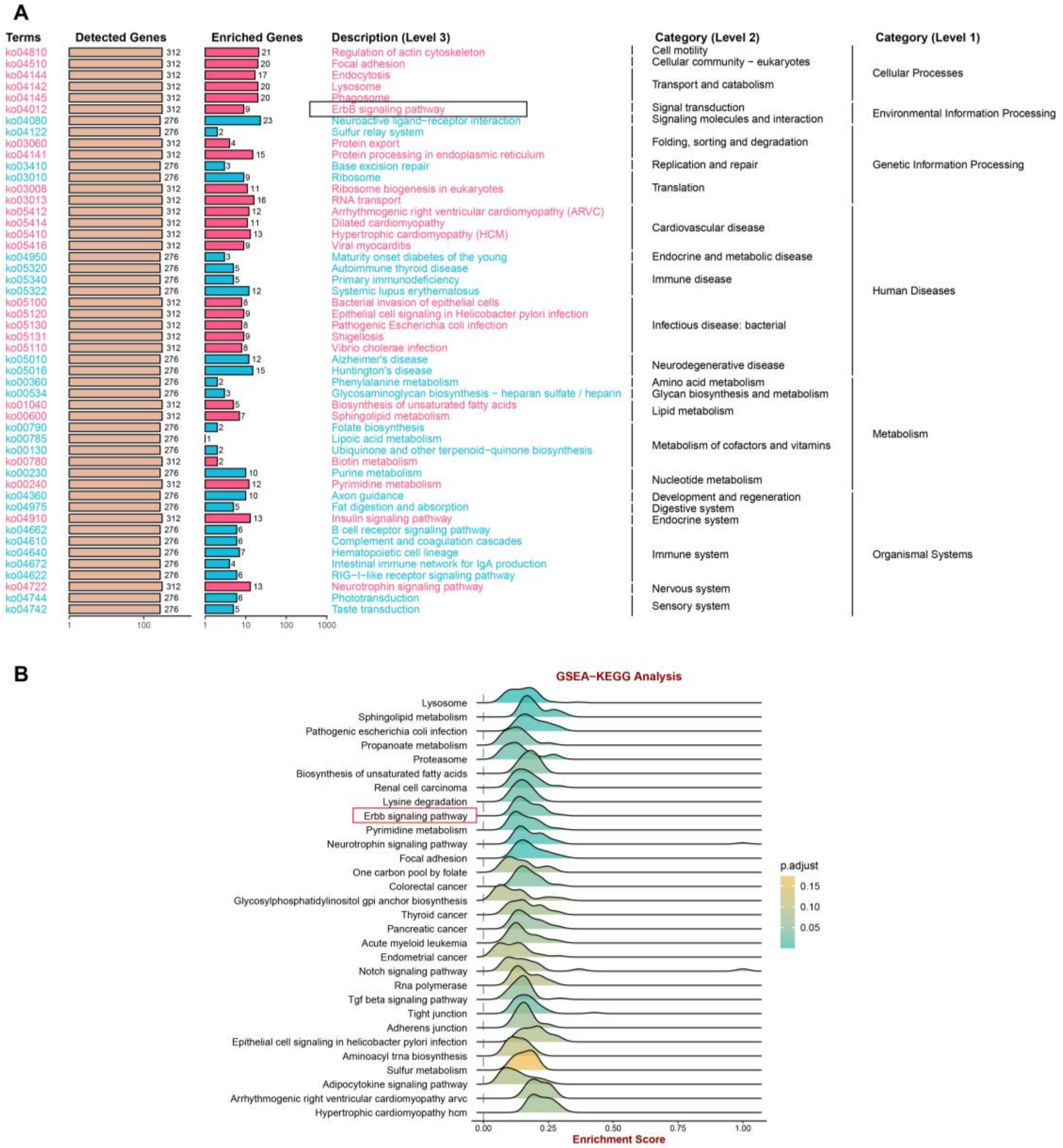

Supplementary Figure. 3 (A) Kyoto Encyclopedia of Genes and Genomes (KEGG) and **(B)** Gene Set Enrichment Analysis (GSEA) based on TCGA-LUAD indicated an association between PSEN1 and Erbb signaling using BEST database (https://rookieutopia.hiplot.com.cn/app_direct/BEST/).
